# Long-Distance Signals Are Required for Morphogenesis of the Regenerating *Xenopus* Tadpole Tail, as Shown by Femtosecond-Laser Ablation

**DOI:** 10.1371/journal.pone.0024953

**Published:** 2011-09-16

**Authors:** Jessica P. Mondia, Michael Levin, Fiorenzo G. Omenetto, Ryan D. Orendorff, Mary Rose Branch, Dany Spencer Adams

**Affiliations:** 1 Department of Biomedical Engineering, Tufts University, Medford, Massachusetts, United States of America; 2 Department of Biology and Center for Regenerative and Developmental Biology, Tufts University, Medford, Massachusetts, United States of America; Instituto de Medicina Molecular, Portugal

## Abstract

**Background:**

With the goal of learning to induce regeneration in human beings as a treatment for tissue loss, research is being conducted into the molecular and physiological details of the regeneration process. The tail of *Xenopus laevis* tadpoles has recently emerged as an important model for these studies; we explored the role of the spinal cord during tadpole tail regeneration.

**Methods and Results:**

Using ultrafast lasers to ablate cells, and Geometric Morphometrics to quantitatively analyze regenerate morphology, we explored the influence of different cell populations. For at least twenty-four hours after amputation (hpa), laser-induced damage to the dorsal midline affected the morphology of the regenerated tail; damage induced 48 hpa or later did not. Targeting different positions along the anterior-posterior (AP) axis caused different shape changes in the regenerate. Interestingly, damaging two positions affected regenerate morphology in a qualitatively different way than did damaging either position alone. Quantitative comparison of regenerate shapes provided strong evidence against a gradient and for the existence of position-specific morphogenetic information along the entire AP axis.

**Conclusions:**

We infer that there is a conduit of morphology-influencing information that requires a continuous dorsal midline, particularly an undamaged spinal cord. Contrary to expectation, this information is not in a gradient and it is not localized to the regeneration bud. We present a model of morphogenetic information flow from tissue undamaged by amputation and conclude that studies of information coming from far outside the amputation plane and regeneration bud will be critical for understanding regeneration and for translating fundamental understanding into biomedical approaches.

## Introduction

**Figure 1:**
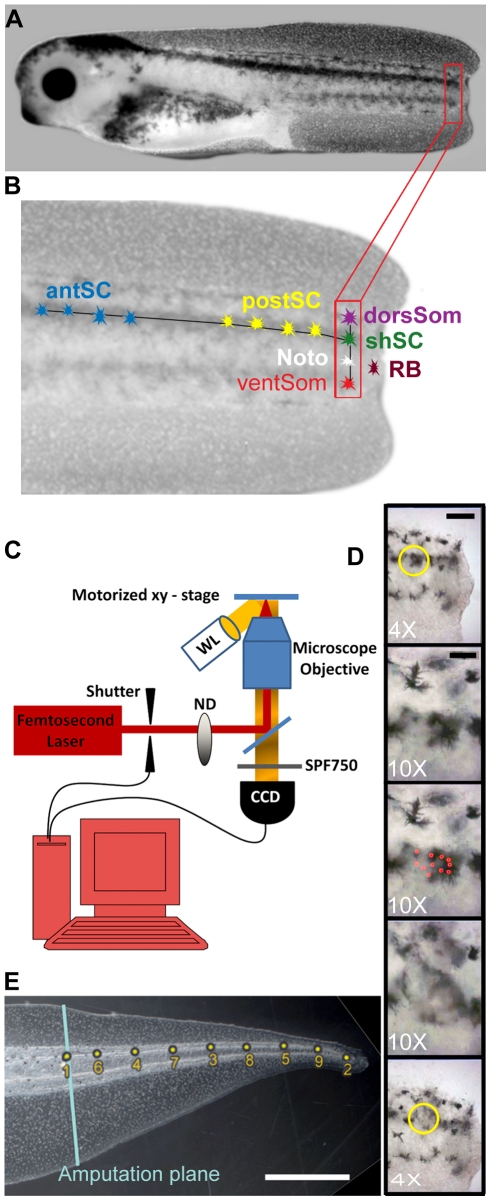
Illustration of the tadpole tail and techniques employed. (**A**) A stage 40 *Xenopus* tadpole shown 2–3 hours after amputation of one-third to one-half of the tail. (**B**) Enlargement of tail in A showing the regions targeted by the laser. Along the DV axis are the dorsal somite (dorSom); shoulder spinal cord (shSC), notochord (Noto), and ventral somite (ventSom). Along the AP axis of the spinal cord are anterior spinal cord (antSC); posterior spinal cord (postSC); and shoulder spinal cord (shSC). Scale bar = 250 µm (**C**) Schematic of laser targeting setup. Pulses from an fs Ti:sapphire laser pass a shutter and neutral density filter (ND) before entering an inverted microscope housing a dichroic mirror (DM), microscope objective, and short pass filter (SPF). The tadpole sits atop a motorized x-y stage and is illuminated by white light (WL). The computer controls the shutter open/close duration and stage, while synchronously monitoring the specimen with a CCD camera. (**D**) Example of the five images that were recorded for each tail using 4× and 10× microscope objectives. Top to bottom they are: low magnification image before laser treatment, scale bar = 150 µm; high magnification image used to focus and aim, scale bar = 50 µm; high magnification record of the location and number of laser insults; high magnification image of laser damage; low magnification image of damage. (**E**) Image of a regenerated control tail including the position of the amputation plane (blue line) and the positions of the nine landmarks used for the Geometric Morphometrics analysis. Scale bar = 1 mm.

Tails of tadpoles of the Anuran *Xenopus laevis* are complicated appendages that have recently become an important model for the study of vertebrate regeneration [1], [2], [3], [4], [5], [6], [7]. The process is under investigation due to the tremendous biomedical potential of techniques that might induce limb and spinal cord regeneration in humans, since tadpole tails appear to regenerate by tissue renewal [8], as mammals do, and they offer the opportunity to study endogenous mechanisms of regeneration as well as to attempt improvement of regenerative ability during non-regenerative stages. We have previously explored the role of H^+^-flux during tail regeneration [9]; in that work evidence was presented for the presence of a patch of depolarized cells found anterior to the amputation plane, in an area called the shoulder, that is also characterized by the appearance of disorderly melanocytes, pigment cells known to be highly sensitive to the electrical properties of nearby cells [10], [11]. A similar patch of depolarized cells has also been found in the shoulder region of regenerating *Axolotl* (Urodele) tails [12]. While most studies focus on the role of cells at or near the regeneration bud, (Fig. 1B) [13], [14], [15], the discovery of these more anterior depolarized cell patches, as well as other results, suggest that necessary signals come from further away ([16] unpublished observations). Indeed there are older experiments indicating the existence of regeneration-regulating signals that act over long distances and require the spinal cord including signals that come from as far away as the brain [17], [18], [19], [20]. The requirement for an intact spinal cord has most recently been tested by surgical excision [4]. To learn more about the role of different cell populations and long-distance signaling during tail regeneration, we decided to do ablation studies.

Unlike many classical ablation techniques, which use scalpels and needles, laser ablation offers superior aim and resolution. Recently, femtosecond (fs) Ti: sapphire lasers have emerged as a useful micro-dissection tool because they provide tight 3D spatial confinement and hence less collateral damage than other lasers [21]. Also notable is the ability of fs laser insults to kill with single cell accuracy. The utility of fs lasers has been demonstrated in chicks, pigs, zebrafish, *Drosophila*, and *C. elegans*, [22], [23], [24], [25], [26], [27]. However, despite the importance of *Xenopus* as an experimental system with an important history of ablation experiments, laser ablation has not been tried on these embryos. We decided to test the utility of fs lasers in *Xenopus* in the context of answering long standing questions about signaling during tadpole-tail regeneration.

In this manuscript we compare regeneration of the amputated tail with and without laser ablation of cells in the regeneration bud at specific locations along the dorsal-ventral (DV) axis and along the anterior-posterior (AP) axis of the spinal cord. Since most of the tadpole tissue is transparent to our laser wavelength of 810 nm, we focused the laser beam at pigmented cells (melanocytes) that can absorb the laser energy and hence transfer heat to nearby cells, a technique also know as selective photothermolysis [28]. We found that ablating melanocytes located near the spinal cord, up to 24 hours-post-amputation (hpa) caused malformation of the regenerated tail. To explore questions about long-distance signals, we compared the effect of laser-induced, melanocyte-mediated, spinal cord damage at different positions along the spinal cord's AP axis. We found that the more anterior the damage, the greater the effect on regenerate morphology. To quantify the changes in morphology, we employed the techniques of Geometric Morphometrics [29]. This allowed us to rigorously define and describe tail shape, an advantage over categorizing by eye because: (a) investigator bias was greatly reduced; (b) highly complicated shapes could be described without oversimplification; (c) much finer differences between shapes could be detected. Our results confirmed experimental observations and showed that (1) damage to the spinal cord causes changes to the morphology of the regenerated tail; (2) counter to the expectation that regeneration is guided largely by activity at the amputation plane, we found that the more anterior the damage to the spinal cord, the more severe the effect on morphogenesis of the regenerating tail; (3) damage at two different AP levels causes malformations that are qualitatively different from the effects of damage at either site alone. We propose a model of the spatial properties of morphogenetic information and how it affects normal regeneration.

## Materials and Methods

### *Xenopus* husbandry and tail amputation

Embryos were generated and gathered according to standard techniques [30] and in strict accordance with the Guide for the Care and Use of Laboratory Animals of the National Institutes of Health. The protocol was approved by the Institutional Animal Care and Use Committee, Tufts University (Permit Number: 2008-08). Amputation and laser treatment was performed under tricaine anesthesia, and all efforts were made to minimize suffering. At stage 39 to 40, animals were anesthetized with 1.5% tricaine and, once movement ceased, their tails were amputated using a fresh number 10 scalpel blade, between two-thirds and one-half of the distance from trunk to tail tip. The cuts were made perpendicular to the dorsal axis of the tail (Fig. 1A). After a few minutes, the tadpoles were returned to fresh 0.1× Modified Marc's Ringers (MMR) and transferred to the laser lab. Animals to be treated at 48 hpa were maintained in 0.1× MMR at 22°C for 40 hours before transfer.

For exposure to the laser, *Xenopus* embryos (ranging from 2–48 hpa) were placed in the glass depression of a 35 mm FluoroDish (World Precision Instruments; store.wpiinc.com) filled with ∼0.5 ml 1.5% tricaine in 0.1× MMR. The animals were oriented sideways, to present a lateral view of the tail. They were then held in position by a cover slip and positioned so that the laser was aimed at a melanocyte. Between 1 and 20 insults were delivered, with the laser focused first on the middle of a melanocyte, with subsequent insults being delivered to the smaller pigmented areas that form as a result of the first insult breaking apart the melanocyte. Immediately following exposure, tadpoles were prepared for histology or returned to 0.1× MMR and maintained for 8–10 days at 22°C. Once control tails had regenerated, controls and treated tadpoles were anesthetized and photographed.

Three regions, comprising subsets of seven areas of the tail, were targeted: (1) the regeneration bud (RB); (2) the shoulder, including the dorsal somite (dorsSom), shoulder spinal cord (shSC), notochord (noto), and ventral somite (ventSom); and (3) the spinal cord, including the anterior spinal cord (antSC), the posterior spinal cord (postSC) and the shSC (Fig. 1B).

### Laser Ablation

The optical setup used for laser ablation of *Xenopus* tadpoles is shown in Fig. 1C and described in detail in [31]. Pulses with center wavelength of 810 nm, repetition rate 80 MHz, and pulse width of 120 fs, were generated from a Ti:sapphire oscillator (Spectra Physics: Tsunami). The average pulse power at the sample was varied between 100 mW-750 mW using a neutral density wheel. Included in the beam path was a shutter controller (Thorlabs: SC10) to limit the number of pulses incident on the specimen at one time. The pulses were focused onto the specimen using an inverted microscope (Olympus Microscopes: IX71). The focused beam was slightly elliptical with a measured (full width half max) spot size of 2.6 µm×3.4 µm after the 10× microscope objective. The petri dish holding the tadpole sat on top of a motorized x-y stage (Ludl Electronic Products) and its motion was monitored in real-time with a CCD camera. A custom computer interface (NI- LabVIEW™) was designed to move the stage, control the shutter duration, and record the target location (Fig. 1D).

The laser power and the shutter duration were varied to determine useful parameters, defined as settings that caused visible damage while minimizing the number of cavitations bubbles and any damage that caused bleeding or tissue loss through damaged skin. After varying the shutter open time from 10 ms to 1 s and power from 100 mW to 750 mW, we settled on a shutter duration (Δt = 200 ms) and average laser power (P_avg_ = 205 mW). We defined an insult as this dose of laser energy.

### Morphometrics

To position the anesthetized tadpoles with a lateral view presented to the camera, they were gently held by a staple that had been bent so as to cover without crushing the tail. Tails were photographed using a Nikon AZ100 with attached QImaging CD camera controlled by QCapture. Landmarks were placed on digital images using ImageJ [32] (http://rsbweb.nih.gov/ij/). Nine landmarks were used to describe each tail (Fig. 1E, Supporting Information S1). The first two landmarks were placed over the spinal cord at the plane of amputation and at the distal most tip of the notochord. The other seven marks were semilandmarks: the third landmark was placed over the spinal cord halfway between the first two, as determined by eye. The other six landmarks were placed by successive iterations of the halfway placement. When the 209 useable images had each been marked, the x and y coordinates of the marks along with hpa, position of the target, number of insults, laser power, and position of the amputation plane were compiled in excel and imported into MorphoJ [29]. MorphoJ was used to perform the operations needed for morphometric analysis, including calculation of centroids, Procrustes fits and distances, Eigenvalues, and canonical variates. The program also performed the canonical variate analysis (CVA) and resampling (permutation) tests, with α = 0.05 (see Supporting Information S1). MorphoJ is freely available from http://www.flywings.org.uk/MorphoJ_page.htm.

### Histology

**Figure 2:**
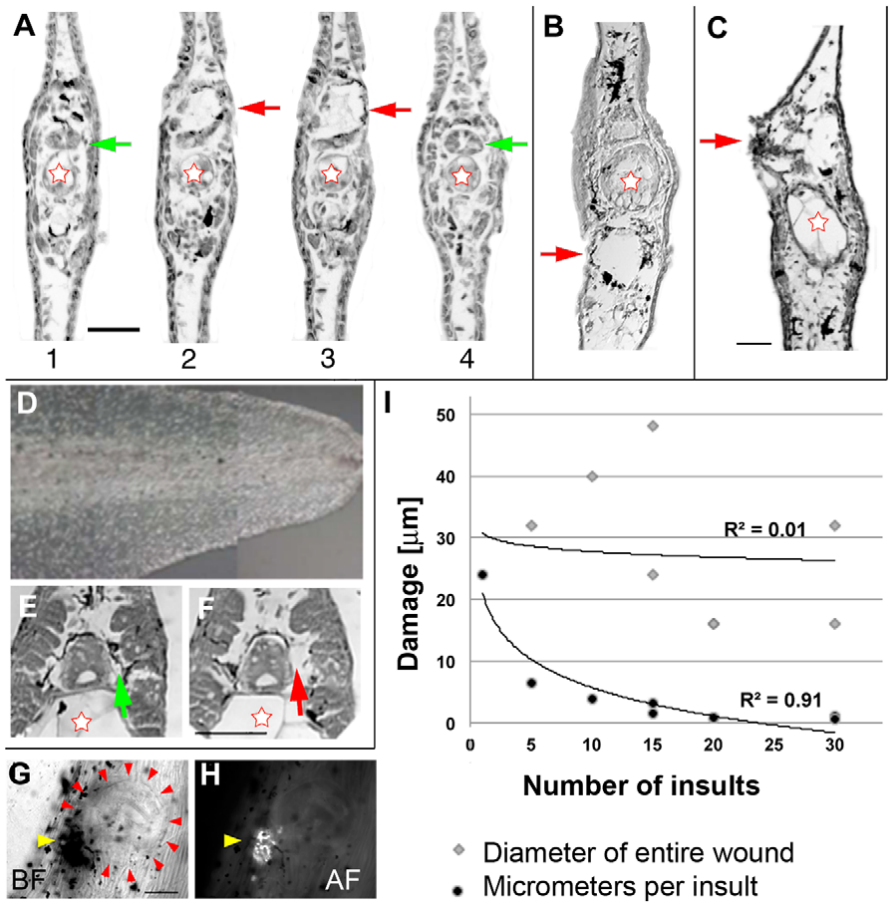
Histology of laser-pulse induced damage. (**A**) Sequential 8 µm sections through a region damaged by a single pulse. The extent of damage was two sections. Red stars indicate the notochord, which was not visibly damaged by the laser pulse. In contrast, the spinal cord was dramatically damaged; green arrows point at the undamaged spinal cord; red arrows point to damage. Scale bar = 500 µm. (**B**) Section showing damage in ventral somite after 15 insults. (**C**) Section showing damage in the shoulder spinal cord after 15 insults. (**D**) Composite of a tail with particularly small melanocytes. (**E**,**F**) Sequential sections from the tail shown in D illustrating that the extent of damage/ablation depends strongly on the size of the melanocyte. In this example, damage is almost entirely restricted to the melanocyte itself (green arrow: undamaged melanocyte; red arrow: ablated melanocyte). (**G**) Image of a wound 8 days after healing at 22°C. The yellow arrowhead points to the cluster of dark pigment spots that appear at the site of laser damage. The red arrows point to the edges of what appears to be a scar formed where the pulses were delivered. Scale bar = 100 µm. (**H**) Epifluorescence (λ_ex_ = 488) image of the wound in G. The arrow points to the autofluorescence emitted by some component of the pigmented cluster. (I) The relationship between the extent of a lesion (gray diamonds) and the damage per laser insult (black circles) as a function of number of insults. The extent of a lesion is calculated by number of section showing damage×8 µm per section.

Immediately after treatment, or at other relevant time points, anesthetized tadpoles were fixed in MEMFA [30] overnight at 4°C then processed for paraffin sectioning. 8 µm sections were stained with haemotoxylin and eosin then photographed using an Olympus BX-61 compound microscope with an Orca AG CC2 camera. The microscope and camera were controlled by Metamorph™. All sections from each sample were examined at 10× for laser damage i.e., loss or disruption of tissue for example between the axial tissues and the surrounding muscle in figure 2A. Note that this is not to be confused with spaces between intact tissues or skin discontinuities caused by tissue shrinkage during fixation.

## Results

### Tissue damage resulting from laser ablation

To characterize the nature of the wounds caused by different numbers of insults, we examined images of histological cross sections from tadpoles fixed immediately after laser exposure. In particular, we focused on tissue damage associated with targeting melanocytes located around the spinal cord, the area that showed the most pronounced changes in the shape of the regenerate. Figure 2A shows four consecutive sections around one target. Since each section is 8 µm, the total damage caused by this single insult was recorded as 16 µm. When multiple insults were delivered as shown in figures 2B and C, a larger area, up to ±50 µm was ablated. Typically, multiple insults of a melanocyte near the spinal cord led to damage of the spinal cord and dorsal muscle (Fig. 2B) while insults to other areas induced damage of the same magnitude (Fig. 2C). The notochord was never seen to be damaged in these sections (red stars in Figs. 2A–C, E). If the insult was delivered to a target where the tissue was very thin, for example near the tip of the tail, the damage could extend all the way through the tail (data not shown). We also noted that the extent of damage seems to be related to the size of the melanocyte. When larger melanocytes were ablated with a single insult, some of the nearby tissue was also compromised (Fig. 2A). On the other hand, with smaller melanocytes only the melanocyte itself was damaged by the insult (Fig. 2D–F). Importantly, we were able to damage internal cells without damaging other cells in the path of the light (Fig. 2F). We also found that as many as eight days later, the site of the wound could easily be identified by a nearby cluster of pigmented spots (arrowheads Fig. 2G), of unknown identity. These spots are below the surface and are strongly autofluorescent at λ_ex_ = 488 (Fig. 2H).

We sought to characterize the extent of damage resulting from different numbers of insults. Sections were obtained from three tadpole tails each having been targeted four times, once each with 1, 5, 15, and 20 insults. Figure 2I plots the total damage (quantified from the number of consecutive damaged sections) as a function of number of insults. We found no correlation between the extent of damage and the number of insults, just a range of sizes between 10 and 50 µm. However, when the damage size was divided by the number of insults, an inverse exponential relationship was found (r^2^ = 0.91). That is, each subsequent insult to pigment from the same melanocyte caused progressively less damage. On average, 15 insults were sufficient to cause damage to the nearby tissue.

### Dependence of regenerate morphology on age

We were interested to know whether laser ablation at different times after amputation would yield any differences in the regenerate. We therefore ablated cells during three different time periods, 4±3 hpa, 24±3 hpa, and 48±3 hpa. These experiments revealed that normal regeneration was sensitive to damage at 4 and 24 hpa. At 48 hpa, however, laser induced damage did not cause morphological abnormalities in the regenerate. The effect of timing was also examined separately for the three subsets of cell types (RB, shoulder, and spinal cord) with the same result, that is, sensitivity at 4 and 24 hours, but not at 48. Therefore, subsequent laser exposure was performed within the first 2–6 hpa unless otherwise stated.

### Ablation of regeneration bud cells and cells along the DV axis

**Figure 3:**
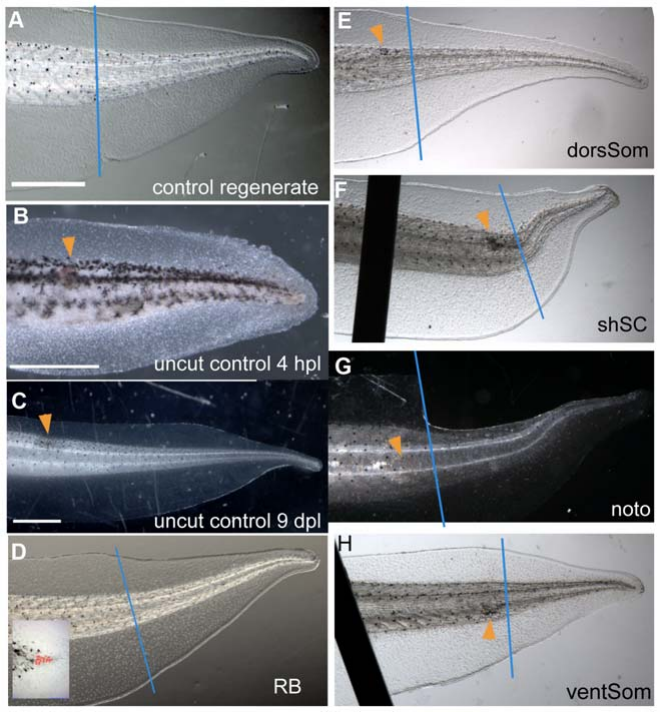
Phenotypes of tails damaged at the regeneration bud and at different levels along the DV axis of the shoulder region. Dorsal is up and posterior is to the right in all images. Typical phenotypes of regenerated tails imaged at stage 48. In all panels, the blue line indicates the amputation plane and the orange arrowhead indicates the position of laser-induced damage. Scale bar = 1 mm. (**A**) Control tail showing the normal shape of the regenerate. (**B**,**C**) Insults to spinal cord show normal development of the tail after 4 hours post laser (hpl) and 9 days post laser (dpl). (**D**) Image of regenerate after ablation of cells in the regeneration bud (RB: see inset). No observable difference was found when compared to the controls. (**E**) Ablation of dorsal somite (dorSom) cells does not affect regenerate shape. (**F**) Ablation of cells in the shoulder spinal cord (shSC) leads to an upward bend. (**G**, **H**) Targeting cells of the notochord (Noto) or ventral somites (ventSom) has no effect on regenerate shape. Dark lines in F and H are the staples used to hold tadpoles flat during imaging.

When tails are amputated and then left undisturbed, regeneration creates a tail very like the tail of uncut controls (Fig. 3A). Likewise, laser damage to the spinal cord of an uncut tail has no effect on the further growth of the tail (Fig. 3B,C). Interestingly, damage to the RB did not cause a discernable change in the regenerate shape (Fig. 3D). Unlike the dorsSom, shSC, noto, and ventSom which usually contain melanocytes, the RB lacks melanocytes and is therefore transparent to our laser wavelength. However, it is possible, with the maximum power used, to ablate this region and still no change in the regenerate was observed.

Because of our interest in the hyperpolarized cells of the shoulder region, which appear at approximately 6 hpa, we examined the effects of ablating different cell populations along the DV axis in the shoulder (Fig. 1A,B). When compared to the controls (Fig. 3A–C) targeted insults to the dorsSom (Fig. 3E) and ventSom (Fig. 3H) caused no observable shape differences of the regenerate. On the other hand, we observed that damage to the shSC caused a pronounced upward dorsal bend of the regenerated tail (Fig. 3F). Less obvious is a slight upward dorsal bend associated with targeted insults to the noto (Fig. 3G).

### Ablation along the AP axis

**Figure 4:**
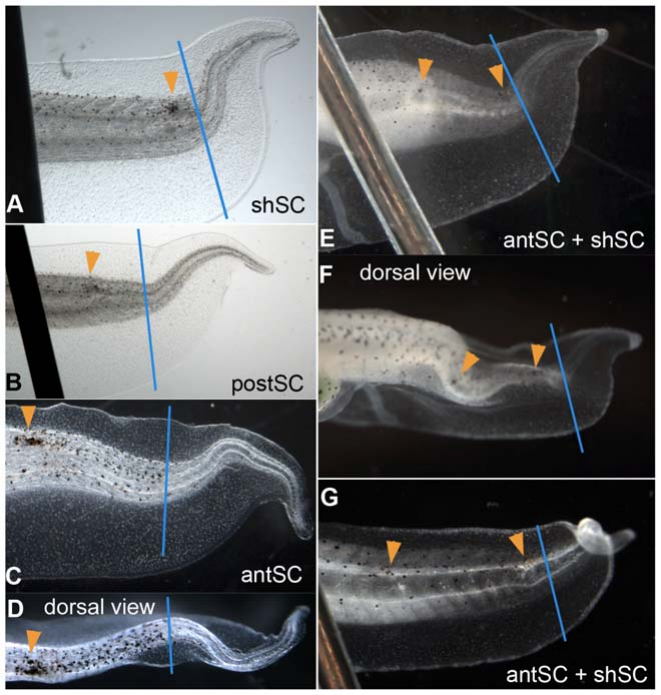
Phenotypes of tails damaged at different sites along the AP axis of the spinal cord. Dorsal is up and posterior is to the right in all images. All tails were damaged at stage 40 and are shown at stage 48. Orange arrowheads point to the location of laser damage; the blue line indicates the amputation plane. Scale bar = 1 mm. (**A**) The typical upward bend phenotype of tails damaged in the shoulder spinal cord (shSC). (**B**) Regenerate for tail damaged in the posterior spinal cord (postSC). (**C**) Image of the more severe phenotype caused by ablation of the anterior spinal cord (antSC). (**D**) Dorsal view of the tail shown in C illustrating the lateral bending of tails damaged in the antSC. (**E** and **F**) A tail damaged at two sites, the antSC and the shSC. This tail shows both characteristics of tails damaged at either shSC or antSC, including a simple upward bend, and LR bending. (**G**) The “pigtail” spiraling at this tail tip is unique to tails that have been damaged at both antSC and shSC.

After finding that ablation of cells in the shSC caused morphological abnormalities in the regenerate, we decided to examine the effect of damage at different AP positions along the length of the spinal cord. We defined two broad areas of the spinal cord, anterior and posterior, where antSC was any position anterior to the midpoint between the amputation plane and the posterior extent of the gut, and postSC comprised positions posterior to the midpoint but anterior to the shoulder (Fig. 1B). We found that ablating cells anywhere along the spinal cord induced gross changes in the morphology of the regenerated tail (Fig. 4). When we compared the effects of ablating cells in different areas (Fig. 4A–D), we observed that the more anterior the damage, the more severe the change to the regenerate morphology appeared to be. In particular, damage to antSC sometimes caused lateral bending of the tail (Fig. 4D) which was not seen in other treatments. Interestingly, the shape change induced by insulting cells in both the antSC and the shSC of one tail, seemed to cause damage that was both more severe than a single insult alone, and qualitatively different from damage caused by insults to either site alone (Fig. 4E–G). Most obvious was the spiraling of the tail tip (Fig. 4G).

### Quantifying regenerate morphologies using Geometric Morphometrics

To quantify shape and thus gain better insight into the differences in regenerative morphology, we used Geometric Morphometrics. This set of techniques allows quantitative descriptions of shapes and analysis of those shapes using multivariate statistics (Supporting Information S1). These techniques are routinely used in the study of evolutionary shape changes [33], [34], and are starting to be used by developmental biologists [35], [36]. Thus we decided to use Geometric Morphometrics to supplement our observational data by quantitatively examining shape changes.

**Figure 5:**
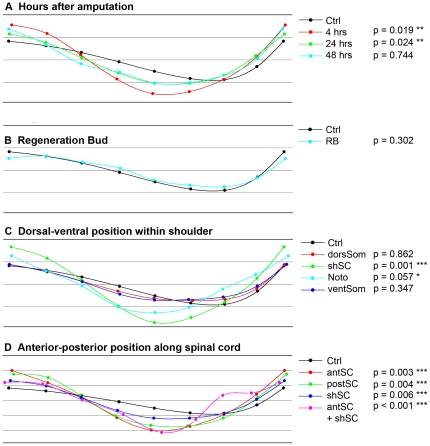
Procrustes profiles of tail morphology after laser treatment. The nine points in all profiles represent the average position of the landmarks used to describe the shape of the regenerate (see Fig. 1E). In all cases the control refers to tails that have regenerated after amputation and all other profiles refer to regenerates after amputation and laser treatment. The numbers next to the legend are the probability that the profile will have that shape. If the P value is <0.05 then we assume the shape is statistically different from the control. The profiles and P values were calculated using Morphometric Geometrics as described in Supporting Information S1. (**A**) Compares the Procrustes profiles for insults to the shoulder spinal cord (shSC) for different hours post amputation (hpa). (**B**) Insults to the regeneration bud (RB) are shown not to affect regeneration. (**C**) For insults along the DV axis, only insults to the shSC and notochord (Noto) were significantly different from the control. Insults to the dorsal somite (dorSom) and ventral somite (ventSom) produced similar regenerates as the controls. (**D**) Profiles of insults along the AP spinal cord axis. The further anterior the damage, the greater the difference from the control. Even larger shape changes were observed in regenerates that had laser damage at two locations, anterior spinal cord (antSC) and shSC. *** indicates p<0.01, ** indicates p<0.05, * indicates p<0.1.

Figure 5 highlights the main results from this analysis. The plots are the control and treated regenerates' Procrustes fits (which can be thought of as average tail shapes; however, see also Supporting Information S1). The nine points in each curve correspond to the landmarks used to describe the profile of each regenerate (Fig. 1E). The numbers next to the legend are the P values (α = 0.05) from permutation tests following the CVA comparing among the treatments, as described in Supporting Information S2. This analysis confirmed all of the above mentioned observations: the sensitive period for laser-induced damage is between 4 and 24 hours (Fig. 5A); laser ablation of the RB in the first 24 hpa did not affect the regenerate (Fig. 5B); the shSC is the most sensitive to laser-induced ablation of the regions along the dorsal ventral axis of the shoulder(Fig. 5C), although the shape variation caused by ablation of notochord cells is close to significant; damage to any AP positions along the spinal cord leads to variation in regenerate phenotype (Fig. 5D). See Supporting Information S2 for complete analyses.

**Figure 6:**
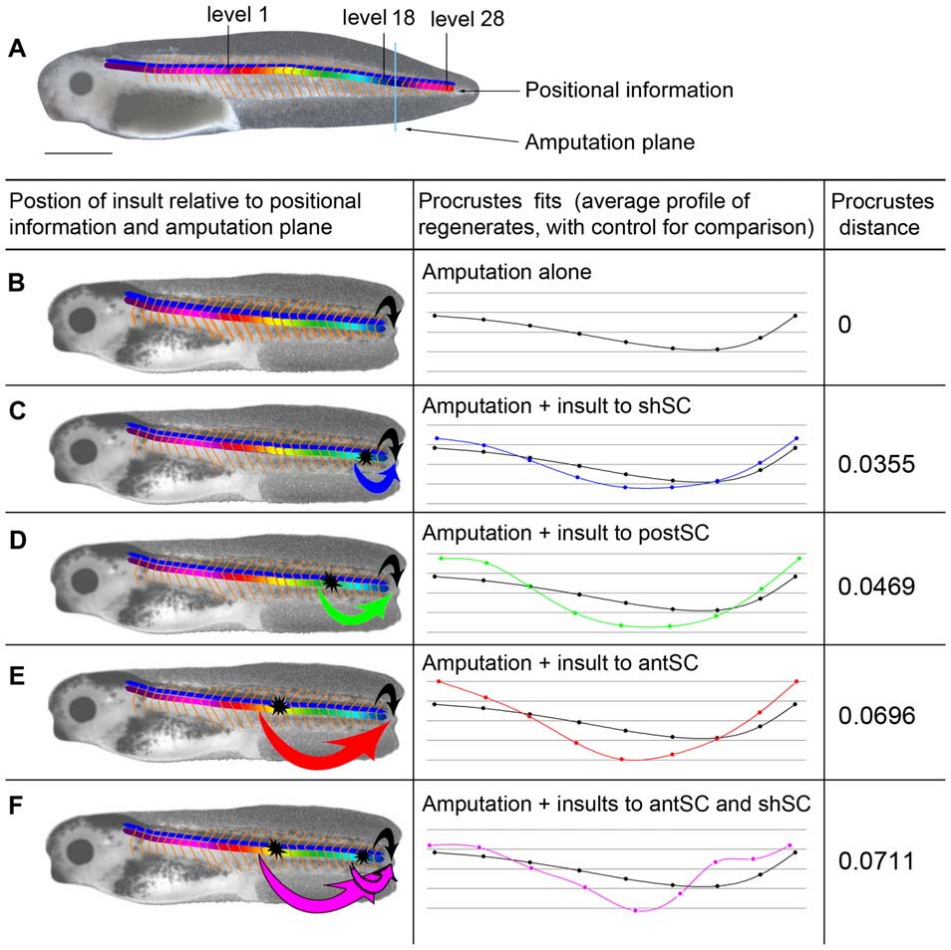
Model of morphogenetic information flow in regenerating tails. (**A**) An intact stage 40 tadpole showing the complete information distribution along the tail. (B–F) A chart of morphogenetic information flow, where the first column shows schematic representations of the information flow, the second column shows the Procrustes fits for tails damaged at specific sites compared with the control, and the final column gives the Procrustes distance between the two shapes. (**B**) A tadpole with an amputated tail. This diagram shows the flow of information (green arrow) that has been activated by the amputation. The origin of this information is the undamaged tail immediately anterior to the amputation plane. This is equivalent to the control situation. (**C**) The flow of morphogenetic information in a tail damaged at one site, close to the amputation plane (upper and lower green arrows). These two sources of information are from similar levels along the AP axis, thus carry morphogenetic information that is essentially the same, leading to only a slight affect on the shape of the regenerate. This is equivalent to damaging the shoulder spinal cord (shSC). (**D**) The information from a more anterior damage site differs more from the information at the cut plane, thus introducing “conflicting” information to the regeneration process, and causing significant variation in shape of the regenerate, like that caused by damage to posterior spinal cord (postSC). (**E**) Damage far from the amputation plane will lead to the presence of morphogenetic information even more in conflict with that at the amputation plane, thus causing a severe affect on shape, like damage to anterior spinal cord (antSC). (**F**) The flow of morphogenetic information in a tail damaged at two sites. This panel illustrates how information from far anterior conflicts with *both* the amputation plane *and* the information from the other damage site, causing different and more severe changes in shape than either site alone, i.e., the greatest Procrustes distance.

Intriguing findings came from further exploration of the CVA of the shape differences caused by damage at different AP segments (Fig. 4, 5D). This analysis yields a measure of the difference between two shapes, called the Procrustes distance. We found that the magnitude of the Procrustes distance between the control shape and the shapes of the treated tails is proportional to the proximity of the insult to the head (i.e. proportional to the inverse of segment number where segment 1 is the most anterior, see Fig. 6). The Procrustes distance between the control shape and the shape of tails insulted at both antSC and shSC was the largest difference in shape that we found (Fig. 6F). Moreover, this variation may have been underestimated because the left-right bending and tail tip curling could not be directly captured in the placement of landmarks on two-dimensional images. We conclude that the closer the damage is to the head, the greater the affect on morphology of the regenerate, while damaging in two different places produces a qualitatively different effect that is also more severe than any caused by insults to one position.

## Discussion

Most current research on tadpole tail regeneration has focused on the processes occurring at or near the site of amputation [7], [9], [15], [37], [38], [39]. These studies are unraveling the genetic and physiological components of the local (caudal) processes that are required for regeneration to occur, and identifying the cell populations that contribute to the tissue in the new tail. In contrast, the work presented here addresses long-standing questions about the role of long distance signals originating in the undisturbed tissue of the regenerating tadpole. We employed two techniques that are relatively new to the studies of development and regeneration: fs laser pulses to ablate cells in a specific area, and Geometric Morphometrics to quantitatively analyze shape. These techniques greatly improved our ability to ablate highly specific and small cell populations, and to detect both qualitative and quantitative differences in morphology.

We found that the damage caused by the laser was similar to that caused by surgical removal of the cord [4] in that laser-induced damage affected the morphogenesis of the regenerate. However, using the laser allowed us to build on the surgical removal technique by providing us better precision and spatial resolution, more importantly, by allowing us to ablate internal cells without damaging the intervening cells. We observed during ablation that the higher the laser power, the greater the damage to the targeted tissue; however, there was no correlation between laser power and the effect on the shape of the regenerate. Also, there was no relationship between the number of insults and the extent of damage (Fig. 2F). There was, however, a strong relationship between the size of the pigmented spot receiving the insult and the amount of damage. For our laser doses, the extent of damage varied from 15–50 µm, on the order of the size of the melanocyte. Thus we conclude that the extent of damage is strongly related to the size of the melanocyte. Therefore, protocols that involve introducing an absorbing dye or marker should account for this dependence on area [25].

Qualitatively and quantitatively we found that the greatest differences in the regenerates were related to laser insults along the AP axis. To understand the signaling mechanism associated with regenerate patterning, we decided to damage the amputated tail at two different locations along the AP axis, at shSC and antSC. If the morphogenetic information were graded, damaging the spinal cord at two positions should lead to changes matching that caused by damage to one or the other single position. That is, both epistasis and the direction of the gradient should be revealed. Our results showed that the shape changes could not be accounted for by a gradient model of positional information. In addition, these morphological differences among the regenerates are not predicted by a simple model of a caudally-derived signal, i.e. a single signal generated at the amputation plane or regeneration bud would influence the regenerate equally no matter where the spinal cord had been interrupted. Therefore, we created a new model to account for these, as well as extant, results.

That undisturbed tissue should contribute to regulating tail regeneration is not surprising. While long-range signals regulating regeneration are still poorly understood, a number of studies have indicated that the factors controlling regenerative processes may not entirely originate local to the blastema. These include cardiac regeneration in zebrafish [40], head-tail determination in planaria [41], and immune system function in amphibian appendage regeneration [42], [43]. In *Xenopus* tadpoles, amputation leads to regeneration of approximately the amount of tail that was removed, therefore there must be a mechanism by which the amount of regeneration is matched to the level of amputation. We propose that information about AP position is encoded with segment identity, and that each tadpole tail segment maintains a marker of its position (possibly *because* it is a structure capable of regeneration). Therefore the positional identity could reside in any segmented component: muscle, peripheral nervous system; vasculature; extracellular matrix, and/or the spinal cord, which we believe to be the strongest candidate (Fig. 6A). Figures 6B–F illustrate how a segment map could affect regeneration. The tissue at or near the level of the amputation plane (“level 18”) sends a signal to the regeneration machinery to grow enough to replace the lost levels, but not to regrow the undamaged levels. When multiple levels are damaged, each sends a different message to the generation mechanism. These partially contradictory signals must act in concert with the other necessary components such as the TGFβ, Bmp, Wnt, FGF, Shh, and Notch signal pathways [44], and with the different cell types, such as the stem cells of the muscles, notochord and spinal cord, that build the regenerate [45].

Consider the effect of the secondary, laser-induced damage as illustrated in Fig. 6C. If the second signal originates at a level close to the amputation plane (postSC), the information about required proliferation and not-required segments will be close to that provided at the amputation plane, thus the combination of the two signals is not too contradictory and can be integrated by the regeneration machinery. As the position of damage, and thus the second positional information source, moves anteriorly relative to the amputation plane, the difference between the normal and secondary signals will increase, making the information provided to the regeneration apparatus more and more conflicting (Fig. 6D,E). Indeed, reading down the table in figure 6, the insult moves anterior and the Procrustes distances increases. This results because the balance of stimulation and inhibition becomes disrupted, with different parts of the regenerating tail receiving different net signals. This explains the differences in relative growth of the dorsal and ventral aspects of the tail that lead to bending. Because the effect of the information conflict is to cause bending in the DV axis, we propose that the information content of the signals concerns growth.

Also consistent with our model of non-graded, non-additive morphogenetic information is the result obtained when two secondary sites of damage were created (Fig. 6F). Not only did that induce the largest quantitative change in shape relative to controls (the largest Procrustes distance from controls), the shape change was also qualitatively different from that caused by damage to either of the two positions singly. Only the doubly damaged tails produced regenerates that spiral into “pigtails.” While we cannot explain this growth pattern, it could be related to influence from the notochord, which is wrapped in-spiraling collagen [46].

The identity of the morphogenetic information is still an open question. A signal dependent on diffusion, such as the classic examples of graded morphogens like Sonic hedgehog, cannot explain the data. Our results require a long-range signal that can cross thousands of cells; thus, diffusion is an unlikely mechanism. Morphogenetic information in the form of a chemical signal could be carried long distances by the neurons in the spinal cord or by the circulatory system. Our histological results suggest that the major dorsal and ventral vessels are not damaged by the laser insults; however, the smaller vessels that feed individual levels were certainly destroyed. The circulatory system as conduit is an interesting hypothesis because it provides both the means of encoding positional information – the loss of the circulation at a particular location along the AP axis – and the means of conveying information about the second site of damage to the amputation plane. However, this hypothesis has never been addressed. Another possibility is that the information is encoded in bioelectrical signals, a possibility that makes particular sense if the spinal cord is the conduit. Again, work on this aspect of regeneration has focused on the caudal end of the regenerating tail [9], [12], [15], [47], thus little is known about the role of bioelectrical signals originating in tissue that is not near the amputation plane. Classical studies in salamanders had shown that innervation is crucial to regenerative ability [48], [49], [50]. However, those data had not demonstrated a role for the nervous system in patterning of the regenerate, and were largely consistent with permissive factors allowing the process to go forward. This is in contrast to our data, which suggest that signals from (or traveling along) the spinal cord are determinative of shape in the newly regenerating appendage.

Hauser published evidence that the source of the information, which requires the spinal cord, is actually in the brain [17]. The regenerate phenotypes he induced by damaging the subcommissural organ in the brain, or the spinal cord at what he termed the base of the tail (equivalent to antSC) match the phenotypes caused by laser ablation at antSC (compare Fig. 3D to Fig. 1b in [17]); it is not clear from the published images if there was left-right bending in those tails. His data suggest that the information is carried by Reissner's fiber, a continuously renewing strand of large-molecular mass, core-glycosylated proteins that starts in the brain and grows down the entire length of the central canal of the spinal cord. It is clear from our histological results that the laser disrupts or destroys the central canal (Fig. 2) thus the hypothesis that this fiber is the information conduit is consistent with our data; it does not, however, provide insight into the AP coding of the information.

### Summary

The potential contributions to medicine promised by a greater understanding of regeneration are truly exciting, and there are an ever-growing number of studies of vertebrate regeneration. Surprisingly, despite the requirement for information to flow from undamaged tissue to the regeneration machinery, most work has focused on understanding only the tissue that is local to the amputation. We expanded the research scope to also include long-distance signals in *Xenopus* tail regeneration. Our method consisted of targeting many individual melanocytes on and inside the tadpole tail with fs pulses from a Ti:sapphire laser such that the absorbed heat damaged cells in their vicinities. We observed that the largest change in regenerate shape occurred along the AP axis where histological sections showed spinal cord damage. Quantifying the resulting shapes using Geometric Morphometrics allowed us to analyze these effects with much greater precision than has been possible with observations by eye. Our results suggest the existence of a long-distance, non-graded signal that affects morphogenesis of the regenerate. This work highlights the critical importance of long-distance signals for normal regeneration, and illustrates the need for more studies on the role of the entire animal, not just the cells that participate directly in replacing lost tissue. Such an approach could lead to better understanding of how to induce regeneration, and thus represents a new approach to the design of biomedical treatments for lost or damaged tissue.

## Supporting Information

Supporting Information S1 **Figure 1.** (**A**) Average Procrustes fits of the four treatments, control, 4 hpa, 24 hpa, and 48 hpa. (**B**) The CVA performed by MorphoJ yielded these CVs, each of which accounts for some aspect of the variation in the Procrustes fits; CV1 accounts for different amounts of bend at the ends, CV2 accounts for different amounts of bending in the middle. The orange and magenta lines are the positive extremes of that component of the shape variation. The black line is the shape of controls for comparison. (**C**) Graph of the 209 procrustes fits (individual points) on a plane defined by the two CVs. The position of the point with the most positive value of each CV corresponds to the orange or magenta line (from **B**) on the axis (thin black arrows). The origin (0,0) of the graph is at the position representing the average of all of the points on the graph. Ovals represent the 95% confidence limits for the means of each group. **Figure 2. Average procrustes fits of control regenerates and regenerates insulted 4 hours after amputation.** The root mean square (RMS) of the distances between corresponding points, represented here by thin black lines, is the difference, or Procrustes distance, between the two shapes.
(DOC)

Supporting Information S2 **Figure 1. Canonical variates and canonical variate analysis of shape differences among control regenerates, and regenerates from tails insulted at different times after amputation.** In all of the analyses, it is clear from looking at the canonical variates that the shape changes induced by laser damage could largely be characterized by changes to the overall bend of the tail (i.e. CV2 in **A**) and changes to the bending of the tip of the tail (i.e. CV1 in **A**). Insults delivered at 4 hours post amputation (hpa) and 24 hpa caused significant changes in shape compared with controls, as seen by the clear separation of the green and red ovals (4 and 24 hpa respectively) from the black oval (ctrl) in **B**. Insults delivered at 48 hpa had no effect. **Figure 2. Canonical variate describing shape change of regenerate due to insults to the regeneration bud.** The change is very subtle, and is not significantly different from control. **Figure 3. Canonical variates and canonical variate analysis of shape differences among control regenerates, and regenerates from tails insulted at four different positions along the dorsal-ventral axis of the shoulder.** (A) The CVs that describe the shape changes are the typical combination of bends in the middle and at the tip of the tail. Regenerates from tails insulted in the dorsal somite (dorsSom) clearly vary a great deal along the CV2 axis, largely due to one tail with an upward turn at the tip (B and D). This datum was examined and is not an outlier (it is not more than twice the inter-quarternary difference away from the median). Despite the influence of this point on the 95% confidence intervals around the mean, the mean shape of the dorsSom group is not different from controls. Tails insulted at the spinal cord (shSC) are highly significantly different from controls, which can be seen in B and C as the clear separation of the green oval (shSC) from the black oval (ctrl) along both the CV1 and CV3 axes. Comparing the yellow oval (noto) to the black oval (ctrl) in C suggests that insults to the notochord may also have an effect. Because of the small number of individuals in the noto group, however, this difference was not statistically significant. The shapes of regenerates after insults to ventSom are not different from ctrl. **Figure 4. Canonical variates and canonical variate analysis of shape differences among control regenerates, and regenerates from tails insulted at four different positions along the anterior-posterior axis of the spinal cord.** (**A**) The typical variation in the overall bend is seen in this group of regnerates. CV1, however, is only found in this analysis, and almost exclusively describes the shape variations caused by double insults. To see the graphical representation of how regenerate shape differences increase as the insult is moved anteriorly, zoom in on **B** and notice the increasing distance between the control mean (black oval) and the red, then green, then blue ovals (shSC, postSC, and antSC respectively). The large size and very different position of the magenta oval (antSC+shSC) illustrates how a double insult leads to shapes that are futher from the control than any single insult, and are in a different part of the graph from the single insults (i.e the variation in shape is in CV1), illustrating that the change is both quantitative and qualitative. The shape difference between ctrl and shSC is in CV3 (**C**) while the difference between ctrl and postSC is visible as differences along the CV2 axis (**B** and **D**) and, to a lesser extent, along the CV1 axis. The difference between antSC and controls is along the CV2 and CV3 axes (**B** and **D**).
(DOC)
