# Supplementary material for: Long-Distance Signals Are Required for Morphogenesis of the Regenerating Xenopus Tadpole Tail, as Shown by Femtosecond-Laser Ablation
Source: PLoS One. 2011 Sep 16;6(9):e24953. doi: 10.1371/journal.pone.0024953 (PMC3174989; doi:10.1371/journal.pone.0024953)
Supplement: Supporting Information S1 — Figure 1. (A) Average Procrustes fits of the four treatments, control, 4 hpa, 24 hpa, and 48 hpa. (B) The CVA performed by MorphoJ yielded these CVs, each of which accounts for some aspect of the variation in the Procrustes fits; CV1 accounts for different amounts of bend at the ends, CV2 accounts for different amounts of bending in the middle. The orange and magenta lines are the positive extremes of that component of the shape variation. The black line is the shape of controls for comparison. (C) Graph of the 209 procrustes fits (individual points) on a plane defined by the two CVs. The position of the point with the most positive value of each CV corresponds to the orange or magenta line (from B) on the axis (thin black arrows). The origin (0,0) of the graph is at the position representing the average of all of the points on the graph. Ovals represent the 95% confidence limits for the means of each group. Figure 2. Average procrustes fits of control regenerates and regenerates insulted 4 hours after amputation. The root mean square (RMS) of the distances between corresponding points, represented here by thin black lines, is the difference, or Procrustes distance, between the two shapes. (DOC) [file pone.0024953.s001.doc]

ABOUT THE GEOMETRIC MORPHOMETRIC ANALYSIS USED IN THIS PAPER

Geometric morphometrics (GM) is a series of data transforms that reduce the number of variables needed to describe shapes. The goal is to reduce the number sufficiently to allow the use of multivariate statistics for data analysis. Different transform techniques are used for different questions; the transforms used for this analysis were:

**1.** **Landmarks**. The first step was to represent the profile of the regenerated part of the tail (the “regenerate”) by nine pairs of X and Y coordinates (units: pixels on a monitor set to 1280x1024 resolution) as shown in Fig. 1E. The first and last landmarks were biologically meaningful positions , namely the amputation plane and the tip of the regenerated notochord. The other seven were semilandmarks, chosen by successively finding the midpoints between the previous points. This digitizing step was performed using ImageJ. A collection of nine points will hereinafter be referred to as a “profile.” The total sample size of this analysis was 209 profiles.

**2.** **Procrustes superimposition**. (This, and all subsequent steps were performed in MorphoJ.) Procrustes superimposition transforms the 209 profiles in three ways. First, it scales each of the profiles to remove differences in size. This requires finding the centroid (the center of gravity) of each profile, which defines the centroid size, (the root mean square of the distances from each landmark to the centroid of that profile). The program then scales each profile so that all the centroid sizes are equal to 1. This step removes most of the contribution of size variation to the estimate of shape variation. Next, each shape is translated across the XY plane so as to put the centroid of each shape at (0,0). Third, each profile is rotated so that the distance between all the corresponding landmarks is minimized. These three processes generate a new set of profiles – the Procrustes fits - that differ from each other *only* in shape, not in size, position, or rotation. This new data set was plotted as the Procrustes profiles in Fig. 5 and 6. The actual data analysis was more complicated and will be described below for the specific example of comparing the effects of insults to tadpoles for different age groups.

**3. Regression of procrustes fits against centroid size**. Procrustes superimposition removes most of the differences in size, but not all. The amount it misses, which is related to allometry (the variation in shape that is due to variation in size) can be calculated and removed. The first step is the calculation, for the Procrustes fits, of the regression score, which is a multivariate measure of shape based on the vector of (X,Y) coordinates of the Procrustes fits. The regression score is then plotted against centroid size. The amount of variation due to size that this regression accounts for is expressed as a percentage of the total variation, thus yielding a measure of the contribution of size differences to shape differences. The rest of the analysis is performed on the residuals of this regression, which are size independent measures of shape variation.

4. **Canonical Variate Analysis (CVA)**. Figure S1.1 shows three different ways of examining the differences in these four shapes. In figure S1.1A, the average of the procrustes fits have been presented together to facilitate comparison by eye, as they are in the paper, but this type of representation lacks important information about shape variation. To capture that variation, the residuals (in this analysis) are further transformed (by multidimensional Fourier transformation), so that the *variations* in shape, rather than the coordinates of the points, become the variables. These new versions of the data are called "canonical variates," and each illustrates one facet of the way the procrustes fits vary. Figure S1.1B shows the two canonical variates (CVs) found by MorphoJ for this example. They indicate that most of the variation among the 209 Procrustes fits can be summarized as differences in either a bend in the middle (CV1) or bending at the ends (CV2). The two canonical variates are then used as axes to generate a plane on which every individual can be graphed. In other words, instead of nine (X,Y) pairs, each Procrustes fit now has one (CV1,CV2) pair. The graph for this example is shown in figure S1.1C.


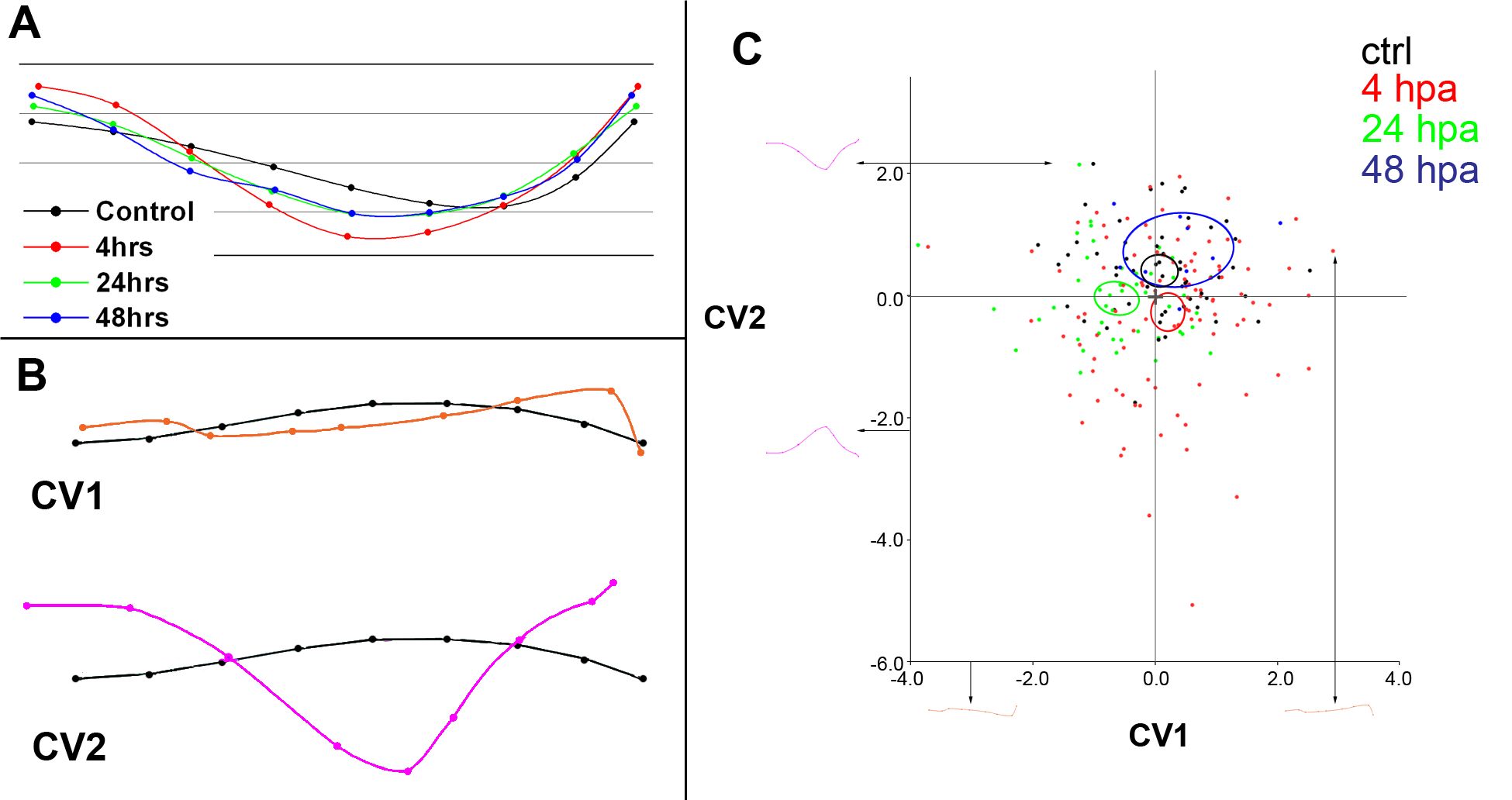


Supplement 1 Figure 1.

The method of representing the data in figure S1.1C makes it clear that the shapes of regenerates insulted at 4 or 24 hpa (red and green) differ from controls in the magnitude of the bend in the middle while regenerates insulted at 48 hpa do not. Also worth noting is that the extra variation is in the negative direction, meaning the shapes of the most affected regenerates are the inverse of the orange line representing CV1 in B, that is, they bend up in the middle.

Two sets of numbers are of interest in the data analysis: the Procrustes distance and P-value of these distances.The Procrustes distance is defined as the root mean square of the distance between corresponding points on two average Procrustes fits (Fig. S1.2).


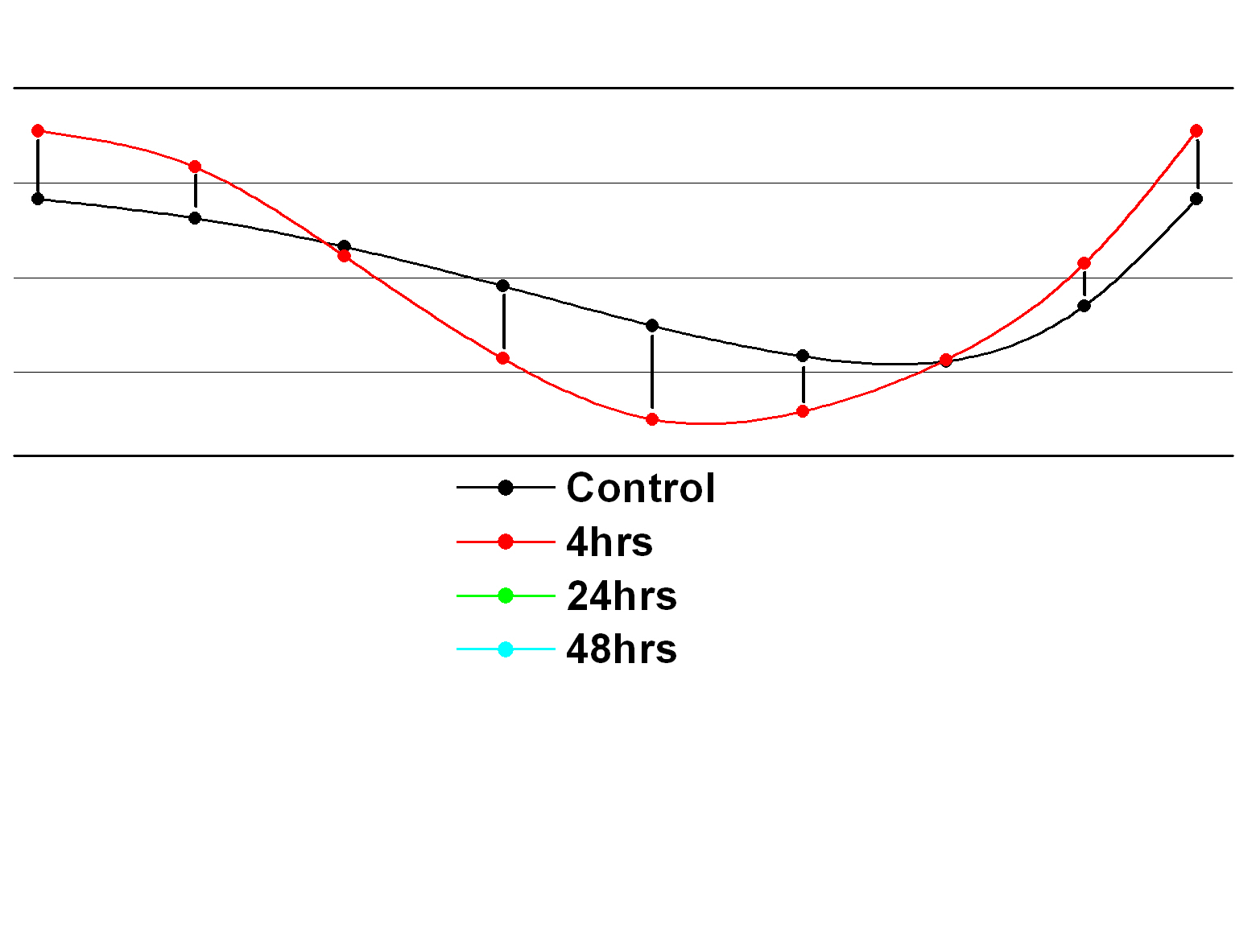


Supplement 1 Figure 2

The Procrustes distances between the control and the other three shapes in this example are:

The statistical analysis comparing the CVs (10,000 rounds of permutation tests) confirms that the shapes of regenerates insulted 4 or 24 hpa are different from controls while those insulted at 48 hpa are not:

| Ctrl to 4 hpa | 0.0317 |
| --- | --- |
| Ctrl to 24 hpa | 0.0286 |
| Ctrl to 48 hpa | 0.0141 |

| **P-values** for Procrustes distances among groups: | | |
| --- | --- | --- |
|  |  | |
| Ctrl vs. 4 hpa | p = 0.0192 * |  |
| Ctrl vs. 24 hpa | p = 0.0238 * |  |
| Ctrl vs. 48 hpa | p = 0.7437 |  |
| * p < 0.05 |  |  |
