# Supplementary material for: Long-Distance Signals Are Required for Morphogenesis of the Regenerating Xenopus Tadpole Tail, as Shown by Femtosecond-Laser Ablation
Source: PLoS One. 2011 Sep 16;6(9):e24953. doi: 10.1371/journal.pone.0024953 (PMC3174989; doi:10.1371/journal.pone.0024953)
Supplement: Supporting Information S2 — Figure 1. Canonical variates and canonical variate analysis of shape differences among control regenerates, and regenerates from tails insulted at different times after amputation. In all of the analyses, it is clear from looking at the canonical variates that the shape changes induced by laser damage could largely be characterized by changes to the overall bend of the tail (i.e. CV2 in A) and changes to the bending of the tip of the tail (i.e. CV1 in A). Insults delivered at 4 hours post amputation (hpa) and 24 hpa caused significant changes in shape compared with controls, as seen by the clear separation of the green and red ovals (4 and 24 hpa respectively) from the black oval (ctrl) in B. Insults delivered at 48 hpa had no effect. Figure 2. Canonical variate describing shape change of regenerate due to insults to the regeneration bud. The change is very subtle, and is not significantly different from control. Figure 3. Canonical variates and canonical variate analysis of shape differences among control regenerates, and regenerates from tails insulted at four different positions along the dorsal-ventral axis of the shoulder. (A) The CVs that describe the shape changes are the typical combination of bends in the middle and at the tip of the tail. Regenerates from tails insulted in the dorsal somite (dorsSom) clearly vary a great deal along the CV2 axis, largely due to one tail with an upward turn at the tip (B and D). This datum was examined and is not an outlier (it is not more than twice the inter-quarternary difference away from the median). Despite the influence of this point on the 95% confidence intervals around the mean, the mean shape of the dorsSom group is not different from controls. Tails insulted at the spinal cord (shSC) are highly significantly different from controls, which can be seen in B and C as the clear separation of the green oval (shSC) from the black oval (ctrl) along both the CV1 and CV3 axes. Comparing the yellow oval (noto) to the black [file pone.0024953.s002.doc]

RESULTS OF CANONICAL VARIATE ANALYSES

(see Supplement 1 for an explanation of Geometric Morphometric analysis)

Effects on regenerate of laser damage delivered at different times after amputation.

N=209

Percentage of shape difference attributable to different size: 6.6%

92% of the shape variation is captured by CV1 and CV2:


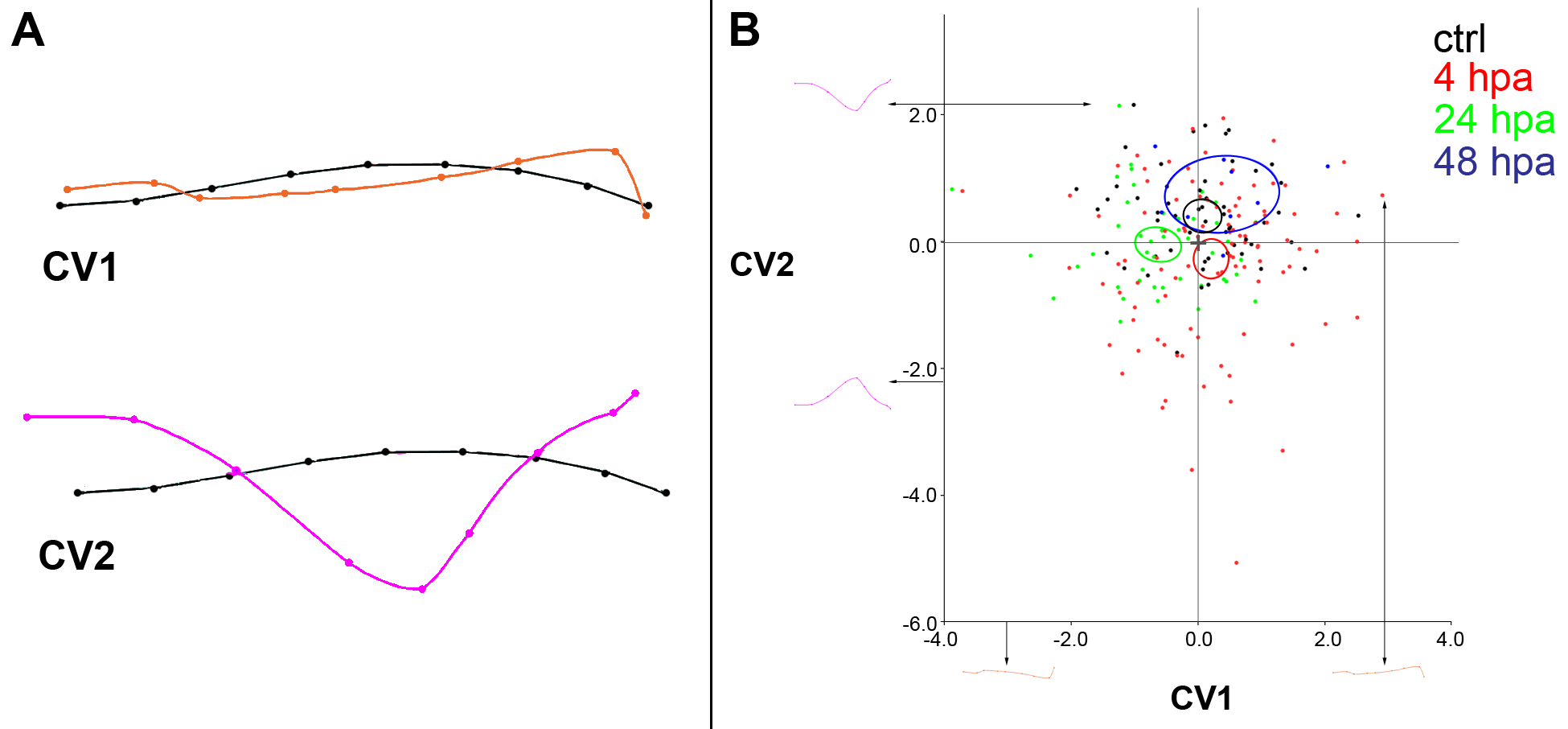


**Supplement 2 Figure 1**.

P-values from CVA with permutation tests (10,000 rounds):

|  | CONTROL |
| --- | --- |
| 4 | 0.0192 ** |
| 24 | 0.0286 ** |
| 48 | 0.7437 |

** p<0.05, * p<0.1

**Effect on regenerate of laser insults to the regeneration bud.**

N=79

Percentage of shape difference attributable to different size: not significant

100% of the shape variation is captured by CV1:


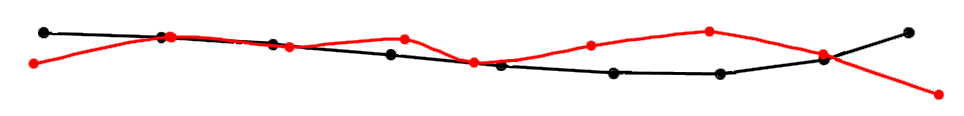


Supplement 2 Figure 2.

P-value from CVA with permutation tests (10,000 rounds): p = 0.3094

**Effects on regenerate of laser insults to the shoulder at different dorsal-ventral positions.**

N=139

Percentage of shape difference attributable to different size: 4.3%

92% of the shape variation is captured by CV1, 2 and 3:


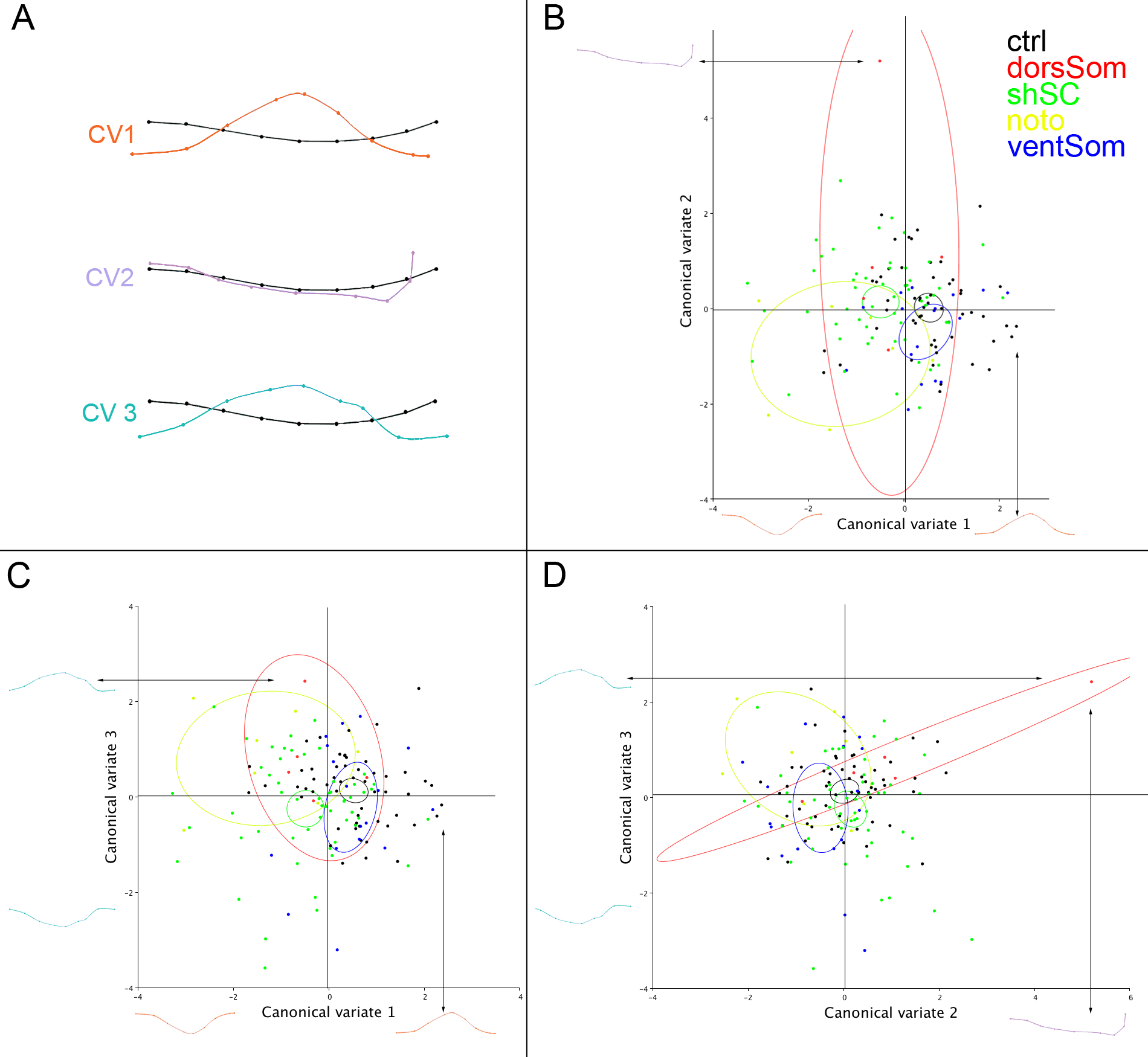


Supplement 2 Figure 2.

P-values from CVA with permutation tests (10,000 rounds):

|  | CONTROL |
| --- | --- |
| Dorsal Somite | 0.8621 |
| Spinal Cord | 0.0011 *** |
| Notochord | 0.0568 * |
| Ventral Somite | 0.3469 |

*** p<0.01, ** p<0.05, * p<0.1

**Effects on regenerate of laser insults to the spinal cord at different anterior-posterior positions.**

N=89

Percentage of shape difference attributable to different size: 6.3%

96% of the shape variation is captured by CV1,2 and 3


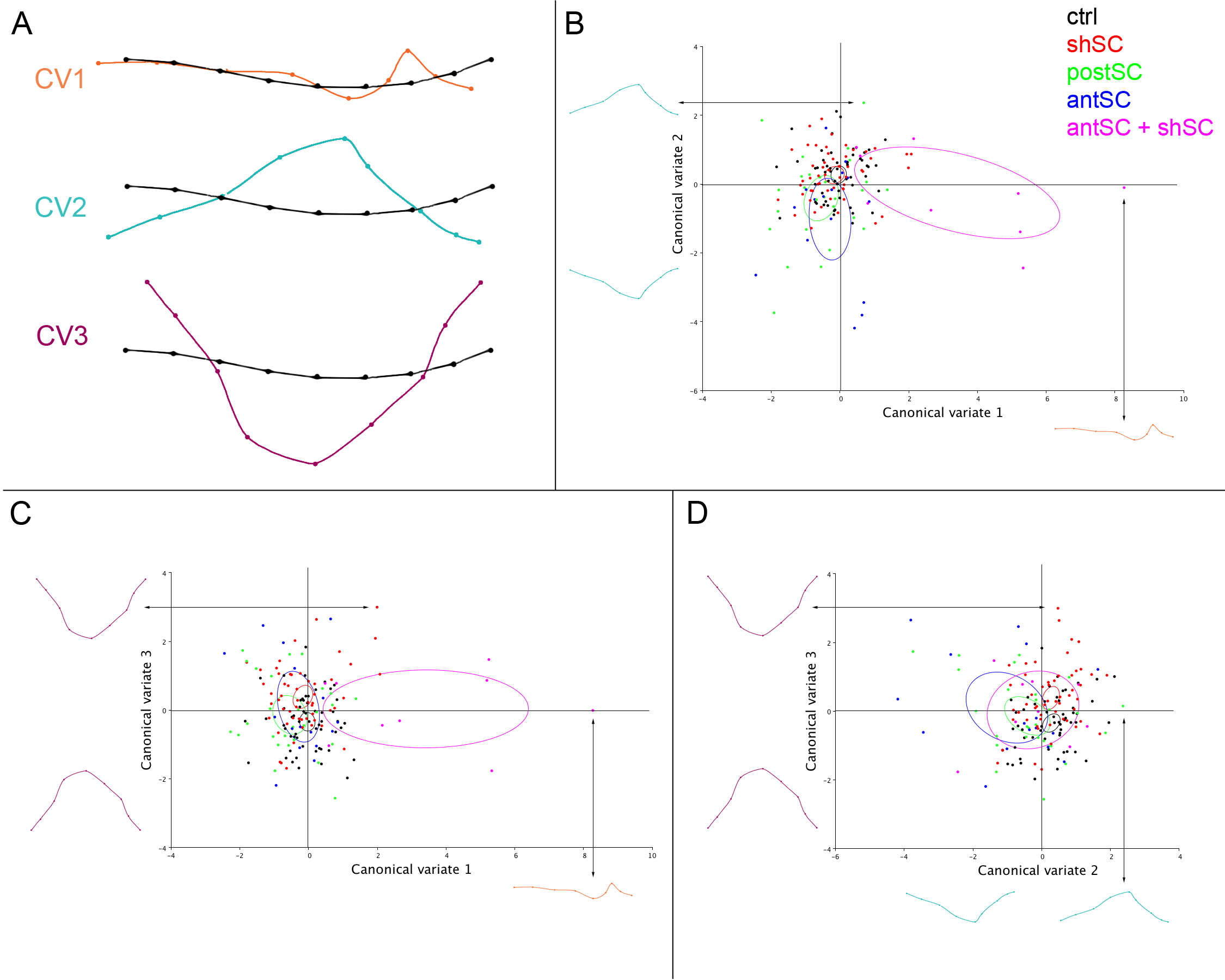


Supplement 2 Figure 3.

P-values from CVA with permutation tests (10,000 rounds) :

|  | CONTROL |
| --- | --- |
| shSC | 0.0062 *** |
| postSC | 0.0039 *** |
| antSC | 0.0033 *** |
| antSC + shSC | 0.0003 *** |

*** p<0.01, ** p<0.05, * p<0.1
